# India’s Frantic Fight Against COVID-19: Rescuing a Broken Healthcare System by Adopting a “Doctor and Patient First” Approach

**DOI:** 10.12669/pjms.38.4.5970

**Published:** 2022

**Authors:** Omar J. Baqal, Amal F. Farouk

**Affiliations:** 1 Omar J. Baqal Department of Internal Medicine, Mayo Clinic, Phoenix, Arizona, United States; 2 Amal F. Farouk, College of Medicine, Alfaisal University, Riyadh, Saudi Arabia

**Keywords:** Indian healthcare system, COVID-19, public health, health policy, patient safety, patient experience

## Abstract

India’s public healthcare system is reeling under the pressure of the COVID-19 pandemic, with the country reporting over 30 million cases and 481,000 deaths by the end of 2021. The rise of the omicron variant threatens to add further strain on a chronically underfunded public health system, which a considerable proportion of the population living in poverty depend on. The pandemic has aggravated the shortage of supplies and capacity, pushing the Indian healthcare system to its breaking point. This write up calls for a major and urgent reform of the doctor and patient experience, achievable through prioritized funding to strengthen healthcare infrastructure, improving job security and satisfaction for healthcare workers, and improving the quality and safety of care delivered to patients throughout the nation. India must build a healthcare system focused on ensuring patient satisfaction and a positive patient experience by offering convenient healthcare access and high standards of care and treatment delivery.

The COVID-19 pandemic has ripped through India, ever since the country confirmed its first COVID-19 case in January 2020, having documented over 30 million cases and over 480,000 deaths by December 2021. Although a stringent nationwide lockdown initially slowed the spread, a widespread laxity in adherence to social distancing measures ensured India quickly descended into chaos, overloading the country’s health care system far beyond its capacity. There were no empty hospital beds, but surely a catastrophic abundance of empty oxygen cylinders.

The total number of cases in the country is vastly underestimated, with experts estimating the actual number of deaths to be four to five times higher than estimated. In April 2021, while Prime Minister Narendra Modi through a televised speech reminded the Indian people that only discipline could help them overcome this nightmare, he was seen holding a massive political rally the next day, cheering on the thousands who had turned up to support him and his political party ahead of the local elections. With the Omicron variant gripping the world and the pandemic spreading faster than ever before, the threat of a deadly third wave is knocking on India’s door. Religious and political gatherings continue largely unmonitored, as Modi was himself recently seen in December 2021, mask-less, addressing a large crowd of tightly-pressed supporters in his Uttar Pradesh constituency of Varanasi.

## Low physician to patient ratio:

In India, while private health care remains financially out of reach for a considerable proportion of the population, government-funded hospitals provide free and highly subsidized outpatient and inpatient services to all Indians. While the private health care sector boasts of generally higher standards of care, the public sector suffers from severe underfunding and a significant disparity in the doctor-to-patient ratio. A research study conducted in rural India concluded that almost 77% of the primary care providers in the area did not have any formal medical training.^1^ Since 2005, the private sector has witnessed most of the healthcare advancements in the country. Hence, it accounts for almost 58% of hospitals and 81% of the doctors in the nation.^2^ Out of the 28 states in the country, only 11 states can boast of a doctor: patient ratio of >1:1,000 (the World Health Organization recommended minimum) while the country averages at 0.9 doctors per 1,000 population. That is a shortage of approximately 600,000 doctors and two million nurses in the public health care sector.^3^ In a country where roughly 68% of the population lives in poverty (less than $2 income/day), most citizens avail public health care services.^4^

## A resource-restricted healthcare system:

The total number of hospital beds in both private and public health care sectors in India is estimated to be around 1,900,000, with 95,000 ICU (Intensive Care Unit) beds and only 48,000 ventilators.^5^ That is a dismal 0.5 beds per 1000 population compared to a world average of 2.9 beds per 1000 population.^6^ The state of India’s crumbling health care exposes a chronic underfunding of the system. India spends just over 3.6% of its GDP (Gross Domestic Product) on health care. The average health care expenditure of OECD countries (Organisation for Economic Co-operation and Development) is 8.8% of their total GDP, with nations like the United States and Germany spending substantially more.^7^

In light of the COVID-19 pandemic, a critical shortage of PPE (Personal Protective Equipment) for frontline health care workers has reportedly forced doctors to use raincoats and helmets instead. As cases rise, doctors and other healthcare workers have faced social discrimination due to the stigma associated with COVID-19 over fears of spreading infection. Despite attempts from the government to curb such events, these incidents are on the rise, with reports of physical assault against doctors and other healthcare workers emerging regularly.

## Addressing physician shortage:

Increasing the number of medical training positions across the country and, more importantly, retaining trained professionals by providing them with suitable job incentives could help address the severe staff shortage. The government can adopt a multitude of policies to achieve these goals and can draw inspiration from their backyard to do so. Cities like Bangalore and Hyderabad, for instance, have attracted a large number of Fortune 500 technology companies to set up R&D labs there. They have done so by providing a series of tax and financial incentives to these companies. As a result, many non-resident Indians have returned home as they can now work on cutting-edge research, progress in their career, and earn wages similar to those paid abroad. The reversal of India’s “technology brain drain” has allowed these cities to transform into global tech hubs and for India to become one of the world’s leading hubs of technology innovation and research.^8^

## Physician reallocation to address lack of healthcare access:

While India’s healthcare sector cannot scale as private technology companies can, there are lessons its policymakers can learn from these experiences. Facilitating greater cross-country collaborations with hospitals in countries like the United States and the United Kingdom, where many Indian-trained physicians emigrate, could greatly benefit its local training programs. It would provide domestic students with access to international medical networks and improved technical knowledge. A poll of 90 Romanian physicians with international work experience showed that 51.1% collaborated with either an individual or institution in Romania during their time abroad.^9^ These alliances have provided Romania with the fortune of building strong cross-country scientific networks, further enhancing their medical training programs.^10^

Studies show that most doctors avoid working in rural areas due to insufficient housing, poor quality of education for children, and poor quality of sanitation, electricity, and transportation facilities.^3^ Thus, further incentivizing employment in rural parts of the nation is needed to address the dire state of rural healthcare. The government must also implement technological advancements to facilitate better access to healthcare, such as telemedicine, especially in the rural population.

## Reinventing the patient experience in India:

Healthcare is fundamentally tied to the patient experience, their emotional reactions, and outcomes. Stemming from a personal recount at a top tertiary-care government hospital in the national capital of Delhi, one of the author’s family members experienced first-hand what it was like to be a patient at such a center. From hospital security to clerical staff, patients were being poorly tended to and simply pushed past. Further, 7-8 patients were being squeezed into the doctors’ cabin at a given time, standing in line with no seating space, and each patient receiving 1-2 minutes of consultation and care amidst and in front of the other lined-up strangers. In addition, with the already meager supplies of COVID–19 resource allocation, the frugal supply of oxygen tanks is being allocated unjustly with states like Delhi, receiving higher per capita allocation of oxygen overlooking several other states with a higher COVID-19 caseload, serving to heighten the disparities across rural communities in an already overburdened healthcare system.^11^

As such, creating a positive patient experience entails several fluid segments, including convenient healthcare access, treatment delivery, and patient satisfaction. Such vital patient interests may seem impossible, weighing down on an already strained healthcare system. Yet, it must be advocated and strongly pushed forth to encourage a positive reform of the Indian healthcare system.

As the world’s fifth-largest economy, India must place healthcare higher on its budget priorities and ensure funding is sufficient and utilized appropriately, by in turn addressing its deeply rooted bureaucratic corruption. We hope India’s healthcare system further evolves into a “doctor and patient first” model, where doctors are enabled to provide the best quality care and citizens receive safe, equitable, and accessible healthcare across the board.

## Authors Contribution:

**OJB:** Conceived, designed and is responsible for integrity of research.

**OJB & AFF:** Did literature review and manuscript writing.

**OJB & AFF:** Did review and final approval of manuscript.
